# Multiple beneficial outcomes of medication therapy management interventions in randomized control trials and non-randomized control trials: A protocol for systematic review and meta-analysis

**DOI:** 10.1097/MD.0000000000031491

**Published:** 2022-10-28

**Authors:** Zhi-Jie Deng, Yu-Feng Ding, Shun-Shun Peng, Lu Wang, An-Hua Wei

**Affiliations:** a Department of Pharmacy, Tongji Hospital, Tongji medical College, Huazhong University of Science and Technology, Wuhan, China.

**Keywords:** beneficial outcomes, medication therapy management, systematic review

## Abstract

Medication therapy management (MTM) service is an effective method to reduce medication-related problems and improve patients’ multiple kinds of outcomes. However, the lack of comprehensive review for MTM services has hindered its development. As a result, we are aiming to evaluate the current benefits of MTM services with multiple outcomes.

**Method:**

An electronic search will be performed for randomized controlled trials (RCTs) or non-randomized control trials (NRCTs) that reported MTM services or pharmaceutical services as interventions from PubMed, The Cochrane Library, Embase, and ClinicalTrial. gov. The odds ratios, mean differences, and standard mean differences and their 95% confidence intervals (95% confidence intervals) will be calculated with fixed or random effect models.

**Results:**

This study will evaluate the multiple benefits of MTM services in clinical endpoints, quality of life, economy, and drug-related problems.

**Conclusion:**

The results will review eligible studies released in the past twenty years and provide more comprehensive evidence of the efficacy of MTM services.

**Ethics and dissemination:**

Ethical approval is not applicable for this study.

## 1. Introduction

Medication therapy management (MTM) service is a kind of pharmaceutical service consisting of medication reconciliation, medical consultation, pharmacist care and other pharmaceutical services to help patients get medical self-management, improve clinical therapeutic effects, enhance medication adherence, cutting down treatment expenditure.^[1]^ Since The Medicare Prescription Drug, Improvement, and Modernization Act of 2003 (Public Law 108–173)^[2]^ requiring that MTM services must be provided for eligible patients with certain chronic conditions through Medicare Part D prescription drug benefits, MTM services have a great development covering almost whole America. To go a step further, American Pharmacists Association and the National Association of Chain Drug Stores Foundation defined 5 core elements of MTM, including medication therapy review, personal medication record, medication-related action plan, intervention and referral, and documentation and follow-up.^[3]^ According to the Fairview Health Services, a large integrated health care system since 1998, MTM services helped over 55% health conditions of patients be improved and create an estimated return on investment of $1.29 per $1 in MTM administrative costs.^[4]^

In 2014, a meta-analysis took a particular literary search and outcome analysis and concluded that MTM interventions in included studies may reduce the frequency of some medication-related problems, including nonadherence, lower the readmission rate and emergency department visits, and costs.^[5]^ But it also mentioned that the evidence was still insufficient for improvement in some outcomes. Though MTM services are evolving nowadays, the situation of lacking scientific evidence is still not changed.^[6,7]^

To have a more comprehensive review of the MTM service’s efficacy, we aim to conduct a systematic review and meta-analysis to evaluate current evidence in randomized controlled trials (RCTs) and non-randomized controlled trials (NRCTs), which will establish more robust evidence and fulfill the gap in the proof of the efficacy of MTM services.

## 2. Methods

This protocol had been registered on PROSPERO (Registered ID: CRD42022349050). And the protocol was developed following the guideline of Preferred Reporting Items for Systematic Reviews and Meta-analyses for systematic review protocols (PRISMA-P).^[8]^

### 2.1. Databases and search strategy

We select 4 mainstream databases, including MEDLINE, PubMed, the Cochrane Library, and ClinicalTrial.gov, as our target to systematically retrieve the records that were published from inception to April 16th, 2022. Full search strategies will be constructed for these 4 databases.

#### 2.1.1. Inclusion criteria and exclusion criteria.

All the studies must be the trial with the control group. RCTs, NRCTs, prospective or retrospective cohort studies, and case-control studies are all eligible for this meta-analysis. Language limitation is set as English. Studies with no control group or written in other languages will be excluded. Studies focusing on the pilot study or feasibility study will be excluded. Trials assessing the efficacy between different pharmaceutical interventions and controlled before-after trials will be excluded too.

The including patients should conform to the following criteria:

1. Adult patients (18 years of age and above).
2. Had at least 1 chronic disease or taken 5 medications or above at the same time.
3. Patients in the intervention group must receive MTM services as an intervention.
4. Other patients who were defined by the article’s author that needing pharmaceutical care are also eligible.

Type of intervention:

Experimental interventions: The trials performed MTM interventions will be included. MTM interventions include medication reconciliation, medication review, and other pharmaceutical care.

Comparator interventions: The control interventions could be usual care, or other basic therapy depending on the participants’ diseases.

Type of outcome:

We divided outcomes into 4 dimensions, clinical endpoints, quality of life, economy, and others.

1. Clinical Endpoints: The outcomes in this dimension can reflect the objective results of interventions. It includes readmission rate, mortality rate, emergency department visit rate, number of adverse events, length of stay in the hospital, and survival time (including the Kaplan–Meier curve).
2. Quality of Life: In this dimension, it includes adherence (including the proportion of days and MMAS-8 scale), medication appropriateness index, EQ-5D (including 3L and 5L), and SF (including 12 and 36).
3. Economy: We will group the cost of hospitalization, medication, and total cost in this dimension to reflect the economical efficacy of pharmaceutical interventions.
4. Others: Satisfaction, medication discrepancies, medication errors, and drug-related problems will be assessed.

### 2.2. Data extraction

#### 2.2.1. Study selection.

Two researchers will independently assess the titles and abstracts of studies we identified by literature search. All the studies will be imported into EndNote 20 (Bld14672) software to screen out the duplicates. The rest relevant studies will be selected according to the predefined inclusion criteria. The full text will be examined to confirm the eligibility if it is necessary. Any divergences will be solved by consensus. Also, we will use EndNote as a records manager in this workflow. A PRISMA flow diagram will be illustrated to show our study selection process.

#### 2.2.2. Data extraction.

The full text of the studies will be read and data will be extracted independently by 2 researchers. The following information will be collected by a predetermined data form generated by Microsoft Excel: basic information (title, first author, year of publication, type of study, location of study); study population (age, sex, sample size, detailed description of participants, diseases); details of interventions and comparison; related outcomes mentioned above and the length of follow-up time. Any disagreement in this process will be solved by consensus or consultation with a third person.

#### 2.2.3. Quality evaluations.

For RCTs, we use the Risk of Bias 2.0^[9]^ recommended by the Cochrane Handbook for Systematic Reviews of Interventions (version 6.3).^[10]^ In this tool, the following aspects will be evaluated: randomization process, deviation from intended interventions, missing outcome data, measurement of outcome, and selection of reported result. Studies will be determined to have low, high, or some concerns levels of bias by these aspects.

For NRCTs, cohort studies, and case-control studies, they will be assessed by ROBINS-I^[11]^ also recommended by the Cochrane Handbook for Systematic Reviews of Interventions (version 6.3).^[10]^ Seven aspects will be considered, including confounding, selection of participants, classification of interventions, deviation from intended interventions, missing data, measurement of outcomes, and selection of reported results. A low, moderate, serious, or critical level risk of bias will be decided by these aspects.

#### 2.2.4. Publication bias assessments.

Studies’ public bias will be evaluated following the instruction of the Cochrane Handbook for Systematic Reviews of Interventions (version 6.3).^[10]^ Studies’ publication bias will be recognized as moderate if the shape of the funnel plot is approximately symmetrical. All the illustrations of funnel plot will be generated by Review Manager (RevMan) 5.4 software.

### 2.3. Statistical analysis

#### 2.3.1. Data synthesis.

Odds ratios and 95% confidence intervals will be calculated in dichotomous outcomes (the patient number of readmission, emergency department visit, mortality, and adverse events). Mean differences and 95% confidence intervals of the continuous outcomes (length of stay, outcomes in quality of life, economy, and others) outcomes will be calculated. To synthesize different scales of quality of life, like EQ-5D-3L and 5l, SF-12 and 36, standard mean differences will be calculated as effect measures to replace mean differences. Also, in the economy dimension we will calculate standard mean differences to avoid huge differences in cost if it is necessary. All the calculations will use fixed- or random-effects models depending on the statistical heterogeneity of studies.

#### 2.3.2. Assessment of heterogeneity.

A χ^2^ test will be used to assess the heterogeneity. *I*^2^ statistic value ranging from 0% to 100% represents the quantity of heterogeneity.^[12]^ A *P* value of χ^2^ test < .05 or *I*^2^ > 50% indicates statistically significant heterogeneity. Potential heterogeneity will be assessed by prespecified subgroup analyses.

#### 2.3.3. Subgroup analysis and investigation of heterogeneity.

Predefined subgroups will be performed to investigate heterogeneity if ample data are available. Division of subgroup will base on the type of studies (RCTs and NRCTs), location of studies (US and Non-US), and whether it is about the chronic disease (Chronic disease, non-chronic disease, and undefined).

A χ^2^ test will be conducted to analyze the subgroup outcomes. If *P* < .05, there will be statistically significant differences between subgroups.

#### 2.3.4. Sensitivity analysis.

To evaluate the robustness of the pooled results, we will carry on a sensitivity analysis. We will use STATA software (version 16) to generate the result of sensitivity analysis for every outcome we evaluated.

### 2.4. Ethics and dissemination

All studies included in this meta-analysis come from public research databases. Ethical approval is not required for this study.

## 3. Discussion

Medication therapy is one of the most usual methods in health care system. Given that improper use of medications may bring catastrophic consequences, rational medication application becomes increasingly significant for patients to help prevent diseases from progression, eliminate disease symptoms and finally regain their health. MTM services are believed to improve patients’ awareness of self-management and limit the frequency of medication ordering errors. Up till the present moment, institutions providing MTM services have distributed all over America and helped >2 million patients.^[4,13]^ However, a comprehensive and systematic review process was vastly required to evaluate the impact of MTM services on outcomes in an era when MTM services are widely spread. Although the results of RCTs are more reliable than NRCTs, NRCTs have a prominent advantage in reflecting real-world data. Thus, in our meta-analysis, we are planning to combine the results of RCTs and NRCTs to get a better understanding of the benefits of outcomes in MTM services.

Multiple outcomes including clinical endpoints, quality of life, economy and others are predefined to acquire an entirely view of MTM. Many results of studies demonstrated a significant effect of MTM. A cluster RCT with follow-up time from 30 days to 12 months indicated that MTM services could reduce the rate of readmission.^[14]^ In many other previous studies, a significant improvement of patients’ quality of life in MTM group over the counterpart was revealed, which were assessed by the EQ-5D and SF scales.^[15–18]^ Additionally, the implement of MTM services contributed to reducing the medical burdens^[15,19,20]^ and the number of drug-related problems^[21,22]^ for patients. Robust evidence could be hardly constructed for the enlightenment of the benefits of MTM since these studies were independent and simply focusing on one or two functions of MTM. What is particularly needed is to synthesize these results to take a further step in understanding the benefits of MTM.

Nevertheless, synthesis of different studies will constitute high heterogenous results. To make our analysis more reliable, we will design a subgroup analysis with research types, study regions and disease patterns as factors to find the resources of heterogeneity. First of all, since lower qualities of results exist in NRCTs, the heterogeneity in synthesized results may be raised by the design of studies. Secondly, the development pace of MTM in different countries showed notable disparities and the US, the first country exploiting MTM services, has more advanced research, which will induce divergences in MTM outcomes in different studies related to US and non-US population. Last but not least, nowadays, MTM services are equally applied to elderly patients with chronic diseases and other patients with any diseases who are in need of MTM services. However, MTM may have a different effect on these different patients. In this way, we defined subgroups of disease to analyze the other possibility of heterogeneity.

There are several possible limitations in our study. Firstly, despite we plan to retrieve the most of mainstream databases to avoid omission of studies as far as possible, the completeness of our search strategies is still deficient since the gray literature databases are not accessed. Secondly, MTM services consist of a variety of pharmaceutical services, which means MTM services conducted in different studies may have differences, thus leading to high heterogeneity and unreliable conclusions. To address this issue, as we planned in our protocol, a sensitivity analysis assessing the robustness of our results will be conducted. Lastly, due to availability for wide indications of MTM services, the study’s object is not confined to a specific disease in our meta-analysis. In future research, we may focus on a specific disease to have a deeper understanding of the precise effects of MTM.

## 4. Conclusion

The results of this meta-analysis will provide comprehensive evidence on the efficacy of MTM services in the aspect of clinical endpoints, quality of life, economy, and others.

## Author contributions

**Conceptualization:** An-Hua Wei.

**Data curation:** Zhi-Jie Deng.

**Formal analysis:** Lu Wang.

**Methodology:** An-Hua Wei.

**Project administration:** An-Hua Wei.

**Software:** Zhi-Jie Deng.

**Supervision:** Yu-Feng Ding.

**Writing – original draft:** Zhi-Jie Deng.

**Writing – review & editing:** Zhi-Jie Deng, Shun-Shun Peng, Lu Wang, An-Hua Wei.
